#  The molecular basis of transient heme-protein interactions: analysis, concept and implementation

**DOI:** 10.1042/BSR20181940

**Published:** 2019-01-30

**Authors:** Amelie Wißbrock, Ajay Abisheck Paul George, Hans Henning Brewitz, Toni Kühl, Diana Imhof

**Affiliations:** Pharmaceutical Biochemistry and Bioanalytics, Pharmaceutical Institute, University of Bonn, An der Immenburg 4, Bonn, Germany

**Keywords:** binding studies, heme binding, heme-protein interactions, heme-binding peptides, heme-binding proteins, structural analysis

## Abstract

Deviant levels of available heme and related molecules can result from pathological situations such as impaired heme biosynthesis or increased hemolysis as a consequence of vascular trauma or bacterial infections. Heme-related biological processes are affected by these situations, and it is essential to fully understand the underlying mechanisms. While heme has long been known as an important prosthetic group of various proteins, its function as a regulatory and signaling molecule is poorly understood. Diseases such as porphyria are caused by impaired heme metabolism, and heme itself might be used as a drug in order to downregulate its own biosynthesis. In addition, heme-driven side effects and symptoms emerging from heme-related pathological conditions are not fully comprehended and thus impede adequate medical treatment. Several heme-regulated proteins have been identified in the past decades, however, the molecular basis of transient heme-protein interactions remains to be explored. Herein, we summarize the results of an in-depth analysis of heme binding to proteins, which revealed specific binding modes and affinities depending on the amino acid sequence. Evaluating the binding behavior of a plethora of heme-peptide complexes resulted in the implementation of a prediction tool (SeqD-HBM) for heme-binding motifs, which eventually led and will perspectively lead to the identification and verification of so far unknown heme-regulated proteins. This systematic approach resulted in a broader picture of the alternative functions of heme as a regulator of proteins. However, knowledge on heme regulation of proteins is still a bottomless barrel that leaves much scope for future research and development.

## Introduction

Heme is a valued, versatile, and vital molecule [1–3]. As a prosthetic group of hemoglobin, heme was initially described by Fritz Ludwig Hünefeld in the 1840s [4]. Two Nobel Prizes awarded to Hans Fischer for the synthesis of hemin in 1930, and to Max Perutz and John Kendrew in 1962, who explored the structure of hemoglobin and myoglobin, honored the eminent role of the molecule which was already recognized in the early 19th century (*cf.* www.Nobelprize.org). However, even though the nature of heme and related physiological processes had been investigated for many decades, another substantial function was only identified in the 1990s: Heme may act as an effector and signaling molecule [5]. Part of this function includes transient heme-protein interactions as found for the human Aminolevulinic acid synthase (ALAS) by Lathrop and Timko in 1993 [6]. Lathrop and Timko are the eponyms of the nowadays broadly used term ‘heme-regulatory motif’ (HRM) that originally described a distinct conserved motif involved in heme-mediated regulation of ALAS [6]. Over the years, the term HRM was refined and meanwhile describes a short amino acid sequence that includes a heme-coordination site and is located on the protein surface [7]. Heme binding to such motifs may alter protein stability and/or function or it can result in the formation of a catalytically active heme-peptide/protein complex [5,8]. Motifs including a cysteine-proline (CP) dipeptide are specified by the term ‘CP motif’ [7]. The latter one is the most prominent representative amongst HRMs [7,9–14]. Today, after more than two decades of research, CP motifs are still the best explored HRMs, nevertheless, there is no doubt about HRMs occurring in much more versatile ways considering also other coordinating amino acids such as histidine- and tyrosine-based motifs. If no functional impact occurs upon heme binding to a protein, the term ‘heme-binding motif’ (HBM) is used to describe a protein sequence stretch that interacts with heme. An intriguing question that arises at this point is, what are the specific requirements for transient heme-protein interactions?

Given the chemical nature of heme, one can easily imagine that a heme-protein interaction occurs at various molecular levels. A transient interaction requires fast heme association and disassociation in order to allow for a situation-dependent response. For regulatory heme, a coordinative bond of the central iron ion to a heteroatom-containing amino acid side chain is observed in the first place. The most prominent heme-coordinating residues are cysteine, histidine, and tyrosine, while methionine and lysine are less frequently found [10]. In addition, hydrophobic interactions and π-π stacking conveyed by the porphyrin ring system as well as electrostatic interactions and hydrogen bonding via the propionate side chains contribute to heme binding and influence binding mode and affinity [10,15]. Therefore, not only the coordinative residue but also the surrounding amino acids are responsible for the decision if and how heme interacts with a specific protein [10,15]. Following the aforementioned initial studies, several heme-regulated proteins have been identified in the last 25 years [5,8]. These heme-regulated proteins take part in diverse biological processes including transcription and translation (e.g. DGCR8 [16], Rev-Erbβ [17] ), ion channel modulation (e.g. hSlo1 [18]), circadian rhythm (Per2 [19]), and cell-cycle regulation (p53 [20]) [5,8]. Moreover, the function of various extracellular proteins such as complement factors (e.g. C1q [21], C3 [22]), and coagulation factors (e.g. FVIII [23]) is altered by transient heme binding. Heme-binding to eminent medical targets as, for instance, the cystathionine-β-synthase [24] or the amyloid β (Aβ) peptide [25], known for its crucial role in Alzheimer’s disease, promoted further interest in potential (patho-)physiological implications of transient heme-protein interplays. Heme interaction with proteins may be of particular interest in the case of amplified hemolysis, for example, as a consequence of vascular injury or action of bacterial hemolysins, resulting in ominously augmented concentrations of biologically available heme. Extending the observations of a heme-mediated change of a protein’s activity and/or stability, a peroxidase-like activity [25] was shown for the Aβ-heme complex raising the question of heme-mediated physiological responses depending on the cellular milieu in specific events, e.g. oxidative stress. On the other hand, heme-mediated protein regulation can be used for therapy as in the case of ALAS mentioned above [26]. In an acute attack, porphyria patients may receive heme preparations that are thought to downregulate ALAS activity in the liver and thereby decrease toxic heme intermediates [26]. Due to the fundamental role of heme, as an oxygen-binding molecule in e.g. hemoglobin and its omnipresence in the blood, it is inevitably necessary to understand basic heme-associated processes as well as heme-mediated protein regulation in order to take appropriate measures as required in the case of risk-bearing pathophysiological conditions.

Even though the awareness of alternative roles of heme increased over the years, a systematic approach to explore the molecular basis of transient heme-protein interactions was missing until 2010, when we started the search for specific interaction patterns, binding motifs, and structural insights concerning the transient binding of heme to peptides and proteins.

### Sequence criteria for heme binding identified by a combinatorial peptide library screening

**Figure 1:**
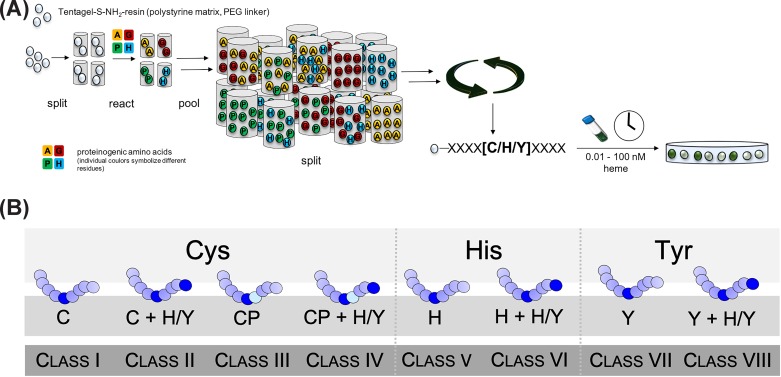
Investigation and classification of heme-binding sequences by means of a combinatorial peptide library (**A**) A combinatorial peptide library X_4_(C/H/Y)^0^X_4_ was constructed in order to investigate sequence specificities of heme-binding peptides. As a result of the screening, a classification system for HRMs/HBMs (**B**) was compiled for cysteine-, histidine-, and tyrosine-based motifs including extra classes for CP motifs [13,29,33,34].

Since short protein-derived sequences (∼9 amino acids) were shown to be suitable to study heme-binding behavior [9,12,27,28], our initial studies included the construction of a combinatorial nonapeptide library based on histidine, tyrosine, and cysteine as heme axial ligand (at position P^0^) as these are the most striking heme-coordinating residues (Figure 1A) [29]. Besides the coordinating amino acid all positions were randomized, and the library was constructed as X_4_(C/H/Y)^0^X_4_ (X: all amino acids except Cys and Met, but including Nle). While methionine was required for technical reasons (special peptide elimination procedure) and thus could not be used at the randomized positions [30], additional cysteines were excluded to avoid intramolecular disulfide formation. After library synthesis, the peptide-bound resin beads were incubated with varying concentrations of heme (0.01 to 100 nM) [29]. Upon incubation a yellow-green color occurred in the case of heme binding and allowed to manually pick the respective beads using a stereomicroscope (Figure 1A).

Sequence elucidation by PED-MALDI-TOF mass spectrometry [30] as well as on-bead automated Edman degradation revealed distinct sequence characteristics [29]. Evaluation of the obtained peptides revealed a predominance of histidine and tyrosine residues (∼40% each) as heme axial ligands over cysteines (∼20%). In addition, corresponding sequence specificities at the termini were identified for all peptides: primarily polar residues as e.g. E, D, Q, N, R, K, H, Y emerged and also, to a lesser extent, hydrophobic amino acids like L, V, F, and Y. Such residues facilitate the interaction with the functional groups of the porphyrin ring. Interestingly, the appearance of additional coordination sites (His/Tyr) was observed in more than 50% of the peptide hits.

The identified hit sequences were analyzed to derive consensus sequences for the different classes of HRMs. To identify potential heme-regulated proteins, a database screening with the consensus sequences was subsequently performed by means of the ScanProsite tool [31] (ExPASy Proteomics server). Several search runs revealed potential heme-binding bacterial as well as human proteins suggesting that the underlying molecular concept is evolutionarily conserved [5,8,32]. Moreover, already published HRMs were evaluated for similarities and sequence characteristics, too. Based on these findings, further fine-tuned consensus sequences were derived and screened as described before. Among the proposed heme-binding sequences, the human dipeptidyl-peptidase 8 (DPP8) was identified as an interesting target protein that was later shown to be regulated by heme [13,29].

### Classifying heme-binding motifs

The findings of the peptide library screening inspired us to develop a classification system for HBMs according to the axial ligand (H, Y, C) (Figure 1B) [33,34]. The observation that more than 50% of the library-derived heme-binding peptides exhibited ancillary coordination sites in close proximity [29], justified a further division of the three classes considering the presence or absence of additional potential coordination sites beyond position P^0^. Thus, a total number of eight classes of HBMs was established (Figure 1B). To allow for profound analysis, suitable peptide representatives of each class as well as proteins carrying the corresponding motif were selected to study their heme-binding behavior with the help of sophisticated spectroscopic methods [13,29,33,34]. The subsequent investigations were based on peptide sequences as model compounds bearing in mind that the gained knowledge was to be transferred to the protein level in following studies.

### In-depth analysis of heme-binding motifs by different spectroscopic methods

**Figure 2:**
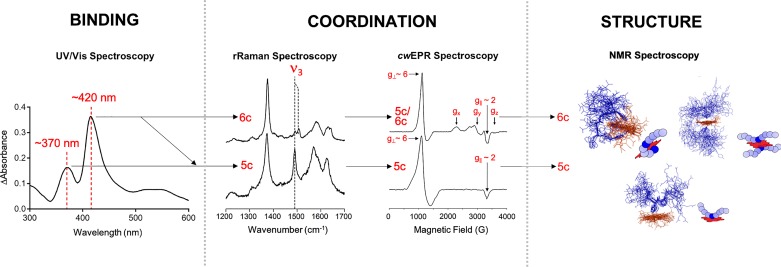
Investigating heme binding to peptides or proteins by various spectroscopic methods Complex formation can be detected by UV/Vis spectroscopy, in particular by a shift of the heme-characteristic Soret-band. Upon binding the heme-iron coordination state, i.e. the occurrence of a penta- and/or hexa-coordinated complex, can be investigated by rRaman and *cw*EPR spectroscopy, focusing amongst others on the ν_3_ band (rRaman) and the signals around *g* ∼ 6 and *g* ∼ 2 (*cw*EPR). The topology and structure of the formed complexes can be clarified by applying 2D- or 3D-NMR spectroscopy [32,33].

The spectroscopic methods applied within our studies complemented each other and allowed to draw a comprehensive picture of the heme-peptide interaction taking place (Figure 2). The fact that relatively large substance quantities are required is common to all of these methods, restricting the practical applicability for molecules with limited availability (e.g. proteins). In the case of nonapeptides, the amount available is usually not the limiting factor, but physicochemical properties affecting solubility, for example, may prevent the implementation of the individual method. Hereafter, a short, simplified insight into the individual spectroscopic methods is given (Figure 2).

Due to the number of conjugated double bonds found in the porphyrin scaffold, heme shows a characteristic absorbance spectrum [35,36]. Especially worth mentioning is the B-band, called Soret-band after its discoverer Jacques-Louis Soret [37]. This band is visible at ∼400 nm. Besides the Soret-band, there are less pronounced Q-bands in the range of ∼450-700 nm [38]. The exact position of the bands depends on the oxidation and spin state of the heme-iron ion as well as the immediate surrounding of the heme molecule [39,40]. As described in the next section, a shift of the Soret-band to ∼370 nm seems to correlate primarily with a penta-coordinated complex, while a shift to ∼420 nm is found for penta- and hexa-coordinated complexes [33,34]. Therefore, the formation of heme-peptide/protein complexes is detectable in the UV/Vis spectrum, in particular with regard to a shift of the Soret-band as is commonly observed for heme binding to amino acid sequences. Moreover, titrations with different concentrations of either interaction partner enable the determination of binding constants such as the dissociation constant *K*_D_.

Once a heme-peptide/protein interaction is confirmed by UV/Vis spectroscopy, the heme-iron coordination state (complex geometry), i.e. a penta- or hexa-coordinated iron ion, is of great interest. The iron ion is bound to four nitrogen atoms of the planar porphyrin ring system while there are two open positions remaining that allow for one or two additional coordinative bonds. These positions can be occupied by ligands possessing sulfur, oxygen or nitrogen atoms such as trifunctional amino acids. Coordination to these ligands will result in the formation of a penta- or hexa-coordinated heme-complex. It is worth mentioning that in hemoproteins the sixth ligand can be a solvent or gas molecule such as water, oxygen, carbon monoxide or nitrogen monoxide, which is usually classified as a penta-coordination with respect to the protein ligand [41]. Methods that allow to determine the coordination state of the heme iron are *resonance* Raman (rRaman) spectroscopy and *continuous wave* electron spin resonance (*cw*EPR) spectroscopy (Figure 2) [42,43]. rRaman spectroscopy is based on laser-induced vibrations (370-430 nm) characteristic for the heme molecule [42,44]. The so-called ν_3_ band is of special interest regarding the iron coordination state, since it shifts according to the coordination occurring during complex formation [44]. The ν_3_ band emerges around ∼1491 cm^−1^ in the case of hemin only (chloride acts as ligand) and a penta-coordinated heme-peptide/protein complex, while in case of a hexa-coordinated complex, the band appears around 1505 cm^−1^ (Figure 2) [44]. Moreover, mixtures of penta- and hexa-coordinated complexes can be detected as a double band [42]. Additional bands such as the ν_7_ band can give further insight into the complex geometry present [45].

*cw*EPR spectroscopy is based on the spin state of the iron ion [43]. Simplified, free hemin as well as a penta-coordinated heme complex exhibit a high-spin state (S = 5/2), while a hexa-coordinated complex results in a low-spin state (S = 6/2) [43]. The respective signals appear at *g*_⊥_ ∼ 6 and *g*_||_ ∼ 2 in the case of penta-coordination, whereas a hexa-coordination leads to the occurrence of three signals with *g*-values (*g*_x_, *g*_y_, *g*_z_) in the range of *g* ∼ 1.5 to *g* ∼ 3 (Figure 2) [34,43]. In-depth information on the structure and topology of heme-peptide/protein complexes can be obtained by applying 2D/3D-NMR spectroscopy (Figure 2) [13]. Changes upon heme complexation are identified by comparing the complex structure to the unbound peptide/protein structure, i.e. evaluation of the chemical shifts of residues before and after heme incubation is necessary [13,46]. NMR spectroscopy of longer sequences is extremely time consuming and challenging primarily due to high structural flexibility [47,48]. Depending on the sequence composition (^1^H, ^13^C) HSQC (heteronuclear single quantum coherence) spectra that are based on the natural abundance of ^13^C are used among other experiments. NMR structural analysis of large peptides and proteins is also possible, yet recombinant expression of ^13^C and/or ^15^N labeled molecules is required, which is usually achieved by adding e.g. ^15^NH_4_Cl and ^13^C_6_-glucose to the respective growth media. The fact that the paramagnetic Fe^3+^ interferes with the surrounding amino acids leads to opposing effects on the intensities and to a broadening of the resonances. Therefore, in many studies other metal porphyrins, e.g. Ga^III^-PPIX, were used instead, which are supposed to interact with peptides/proteins in a similar manner as heme [49–51].

Furthermore, it is possible to use the knowledge obtained regarding sequence requirements for transient heme-peptide/protein interactions to identify HBMs within known heme-binding/heme-regulated proteins. Therefore, we developed a computational tool that allows for the evaluation of potential motifs upon input of the protein sequence.

### Sequence-dependent characteristics of heme-binding motifs

Since the most prominent motifs so far are the aforementioned CP-motifs, our initial studies focused on cysteine-based sequences representing HRM-classes I-IV (Figure 1) [13,33]. Li *et al.* showed that the sole occurrence of a heme-coordination site as well as the presence of a CP-motif does not necessarily result in an interaction with heme [10]. This observation was confirmed within all of our studies, since nonapeptides consisting of a CP-dipeptide surrounded by four respectively three alanine residues did not interact with heme [13,29,33,34]. These findings support the original idea that specific sequence features are required for heme binding. For the cysteine-based motifs (class I-IV) high to moderate binding affinities with *K*_D_ values ranging from 0.40 ± 0.19 μM to 6.36 ± 2.61 µM were determined by UV/Vis spectroscopy [13,29,33]. The cysteine-based sequences with additional coordinating residues (tyrosine, histidine) generally appeared to lead to higher binding affinities [13,33]. The affinities determined seem plausible because a transient interaction requires a fast and uncomplicated association and dissociation of the respective molecules and, in addition, heme is a rather small interaction partner [25,47,52]. The *K*_D_ values obtained are also supported by examples of regulatory heme binding described by other groups [52,53].

Furthermore, evaluation of the observed UV spectra revealed the occurrence of four different kinds of spectra which we categorized as UV-groups I-IV [54]. While a shift of the Soret-band to ∼370 nm primarily represented a penta-coordinated complex as was predominantly found for CP-peptides, a shift to ∼420-430 nm cannot be uniquely assigned to a distinct coordination state although spectra with both maxima frequently exhibited a mixture of penta- and hexa-coordinated complexes as revealed by rRaman and *cw*EPR spectroscopy (Figure 2) [13,33]. In contrast to the CP-peptides, for cysteine-based motifs (without proline) all forms of coordination states were observed [33].

**Figure 3:**
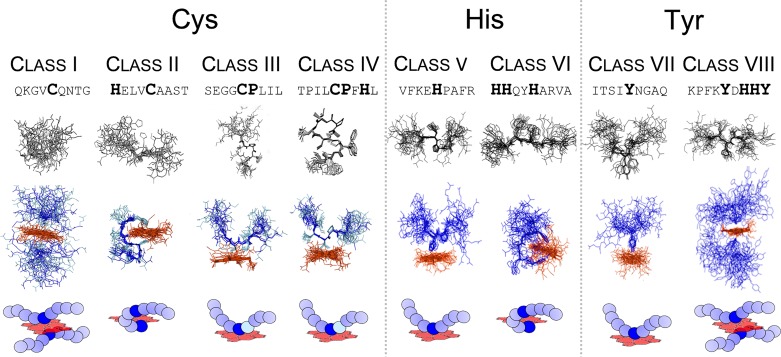
Structural elucidation of heme-binding peptides (classes I–VIII) using 2D-NMR spectroscopy Different binding modes occurred depending on the peptide sequence composition and the formation of penta-coordinated (III, IV, V, and VII) and hexa-coordinated complexes (I, II, VI, and VIII). The latter ones emerged in different forms: a sandwich-like complex including two peptides interacting with one heme molecule (I and VIII) and a loop/clamp-like complex for peptides that exhibit additional coordination sites (II and VI) [32,33].

Structural investigation of selected representatives of classes I-IV by NMR spectroscopy gave insight into the particular role of the proline residue within the CP-motif [13,33]. On the peptide level there was a clear difference between C- and CP-based peptides [33]. The proline residue seemed to reinforce a more defined backbone structure of the free peptide compared to the rather flexible structure of the cysteine-based peptide [33]. Application of heme did not lead to an increase of rigidity in the case of the cysteine-based motifs, however, CP-based peptides showed increased backbone rigidity upon heme binding, in particular in close proximity of the CP-motif. It was found that the proline residue confers a distinct conformation to the subsequent backbone, which - as a consequence - is directed away from the porphyrin ring [33]. NMR spectroscopy also revealed penta-coordinated heme complexes for CP-motifs (class III and IV) independently of the presence or absence of an additional possible heme coordination site [33]. Analysis of the cysteine-based motifs without a proline displayed hexa-coordination for both classes (I and II) (Figures 1 and 3). On the one hand, the cysteine-based peptide with no additional coordination site revealed binding of two peptide molecules to one heme molecule in a ‘sandwich-like’ structure (class I) (Figure 3). On the other hand, for class II (e.g. HXXXC) it was shown that a spacer length of three amino acids is required to obtain a ‘loop-like’ (respectively ‘clamp-like’) hexa-coordinated complex with one coordinating residue being the central cysteine and the other one being a distal histidine residue [33] (Figure 3).

To complete the picture of heme-binding sequences, histidine- and tyrosine-based motifs representing classes V-VIII (Figure 1) were investigated in the same manner as described above [34]. In contrast to the earlier findings for cysteine-based motifs, several sequences did not interact with heme or did not show saturation upon increasing heme concentrations, thereby hampering the determination of *K*_D_ values [34]. The *K*_D_ values determined ranged from 0.24 ± 0.17 μM to 6.25 ± 1.44 μM [34] again revealing high to moderate binding affinities as expected. In-depth analysis of selected sequences revealed that histidine-based motifs usually formed mixed or hexa-coordinated complexes with e.g. an HXXXH-motif exhibiting a loop-like structure, while tyrosine-based motifs predominantly occurred in a penta-coordinated fashion [34]. In the case of an additional coordination site in tyrosine-based peptides no loop formation was found [34]. In general, no increased backbone rigidity as found for the CP-motifs was observed within these studies [34].

Comparing the amino acid composition of the heme-binding sequences revealed a crucial role of the net charge of the nonapeptides. While a negative net charge appeared to inhibit heme interaction, a positive net charge was usually accompanied by a comparably high binding affinity [34]. All the information gained from UV/Vis, rRaman, *cw*EPR, and 2D-NMR spectroscopy revealed insight into the specific characteristics of heme binding to peptides/proteins on the level of primary sequences and secondary structures. In the presented study, peptides served as model system to examine a broad range of primary sequence motifs of heme-binding peptides and proteins. It is worth noting that various other studies have applied peptide-based approaches to investigate heme binding and hemoproteins. These studies addressed functional, structural, stability, and specificity issues of the respective heme complexes [28]. The sequences and secondary structural elements of the examined peptides vary broadly. Whereas some studies use protein-derived sequences, others utilize specifically designed peptides which exhibit desired functional and structural properties. In contrast to the study using nonapeptides, specially designed peptides are often characterized by secondary structures that facilitate distinct functions, e.g. intended heme binding [28,55–59]. Among these are heme-binding multi-stranded β-sheet peptides [55], β-hairpin conformation [56], heme‐Cage β‐Sheet miniproteins [57], as well as helical sequence stretches [59]. Besides basic research these heme-binding peptides and miniproteins are intended for industrial and biomedical applications [58].

Subsequently the established consensus sequences mentioned above were screened against the protein data base ScanProsite tool [31] (ExPASy Proteomics server) aiming to identify so far unknown heme-regulated proteins. The hits obtained were further assessed taking into account the accessibility of the suggested HBM/HRM for heme binding, the protein structure if available, and the possibility to experimentally test the impact of heme on the protein activity. Several potential heme-regulated proteins were identified using this approach. Heme binding to these proteins was verified by spectroscopic methods and the functional impact of heme was shown *in vitro* [13,34,54]. This approach was successful for bacterial proteins such as FeoB [54], chloramphenicol-acetyltransferase (Cat) [32], and hemolysin C [60] as well as human proteins as the aforementioned dipeptidylpeptidase 8 [13]. To summarize the knowledge obtained regarding distinct sequence features of HBMs/HRMs, a procedure that enables evaluation of a protein sequence to comprise HBMs in a stepwise manner was generated (see below). A first assessment of the sequence can be drawn on the basis of the primary sequence. Additional experiments such as UV/Vis spectroscopy will then facilitate pre-evaluation of the heme-binding mode based on general structural features of the complex formed (pre-selection). In order to facilitate the HBM/HRM evaluation process for other users, we recently developed an algorithm termed *SeqD-HBM*. The basics of the *SeqD-HBM* are explained below.

### Evaluating heme-binding capacity of protein sequences using *SeqD-HBM*

A handful of computational tools are available to predict ligand binding to proteins. Some of these have a broader scope of being able to predict the binding of multiple ligands to proteins e.g. *TargetS* [61], whereas other tools are specific for the prediction of heme binding (e.g. *HemeBIND+* [62] and *HemeBIND* [63]). While these tools use complex algorithms and base their predictions on extracting several novel features (e.g. Depth index, Protrusion index, Surface complementarity etc.), the nature of binding usually described is the strong irreversible binding of heme (mostly within a binding pocket) rather than a transient interaction. Moreover, most of these tools rely heavily on the availability of structural information as a basis for their predictions [63]. With this in mind, we introduce a novel tool named “*SeqD-HBM”* for sequence-based identification of HBM using the protein sequence as its primary input (Supplementary Material). The program processes the input sequence through a systematic stepwise validation extracting at each step relevant features from the sequence to produce a tabulated list of the possible heme-coordination sites available for the given sequence. Besides reporting the potential heme-coordination sites (Cys, His, Tyr), the program outputs the 9mer motifs associated with the coordination site and the net charge. Finally, in a column named “comment” useful hints regarding the predicted 9mer motif, such as identification of a CP motif, are provided to the user. This comprehensive output further guides the user to make informed judgments on the nature of heme interaction with the protein of interest.

*SeqD-HBM* in its current stand-alone form has two distinct modes of operation. The *default mode* assumes that the user has no information on the structure related to the input sequence. This consequently means that there is no possibility to determine if a predicted coordination site is “surface-exposed” or “buried”. Prediction of a buried residue as a potential coordination site would be a false positive, defeating the purpose of this tool. To overcome this roadblock, we pass the input sequence through a sequence-based solvent accessibility meta-predictor namely *WESA* (Weighted Ensemble Solvent Accessibility) [64,65]. *WESA* determines the solvent accessibility of each residue in a sequence using an ensemble of five methods: Bayesian statistics, multiple linear regression, decision tree, neural network, and support vector machine. A weighted sum of individual predictions determines the final prediction. This deems *WESA* to be a robust and reliable tool to distinguish between the buried and exposed states of residues and has a published accuracy of 80%. *WESA* is invoked from within *SeqD-HBM* and only those coordination sites that are predicted to be “exposed” are considered for the final tabulated output of the *SeqD-HBM* program.

**Figure 4:**
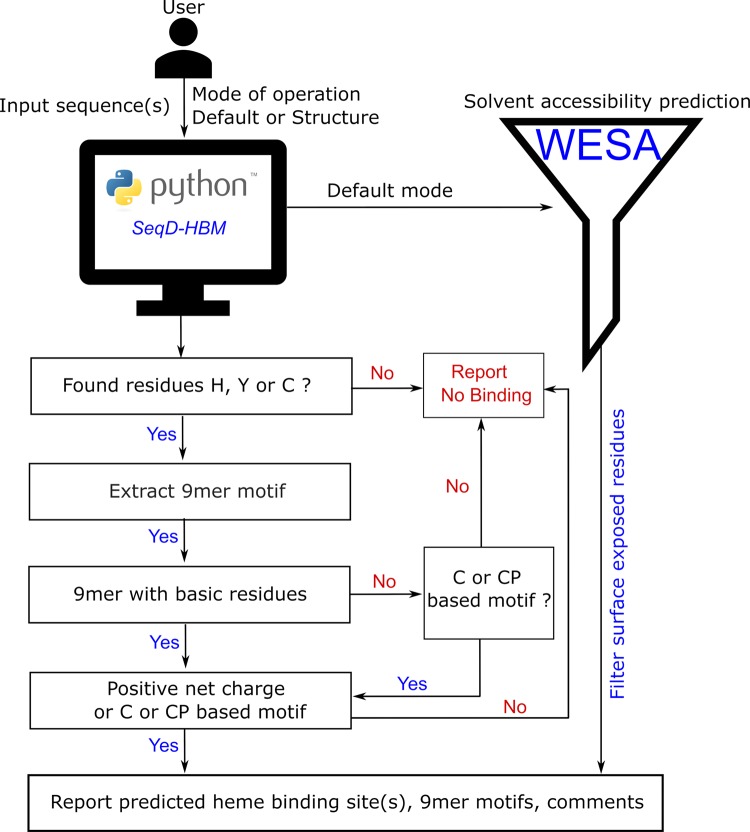
Computational prediction of heme-binding protein sequences Computational evaluation of the heme-binding potential of various peptide/protein sequences based on the knowledge gained from in-depth spectroscopic studies on heme-peptide complexes.

The second mode of operation called the *structure mode* assumes that the user is aware of the structure or is in possession of the structure data of a sequence that is passed as input to *SeqD-HBM.* In this case, *SeqD-HBM* does not invoke WESA and the HBM validation checks are done on every possible coordination site available in the sequence. The user is expected to use the known structural information to manually filter out false positives (i.e. ignore a coordination site prediction if it is known from the structure that the site is a buried residue) from the *SeqD-HBM* prediction. The operation of the application is presented as a flowchart in Figure 4. The implementation and testing details of *SeqD-HBM* are discussed in the Supplementary Material.

## Conclusion

To gain a deeper insight into the molecular basis of transient heme binding, nonapeptides were used as models in order to allow for a global investigation of heme-binding characteristics. More than 200 heme-peptide complexes based on cysteine, histidine, or tyrosine as heme axial ligand have been examined so far using UV/Vis spectroscopy and, in part, methods such as rRaman, *cw*EPR, and 2D-NMR spectroscopy. Evaluation of the data obtained revealed specific sequence features such as a positive net charge of the heme-binding sequence or the existence of hydrophobic residues which have a positive effect on the heme-peptide/protein interplay. Depending on the sequence composition, different binding modes have been observed, e.g. penta- vs. hexa-coordination or mixtures thereof. The knowledge derived from the detailed analysis may first be used to predict heme binding to proteins based on consensus sequences and respective data base searches. Prediction and verification of unknown potentially heme-binding proteins based on such a consensus sequences search has been successful in several cases, i.e. bacterial FeoB, and HlyC. Second, it may be utilized to assess the heme-binding capacity of proteins which were shown to bind heme and to identify HBMs in such sequences. To make the evaluation of heme-binding sequences available to the public, our knowledge was incorporated into the program ‘*SeqD-HBM’* for the determination of HBMs in proteins. The software evaluates the motifs contained in a protein on the basis of the primary sequence and, if possible, takes structural features into account. We expect that such a tool will be useful to decipher molecular details on heme-binding/regulated proteins and in this way support basic research concerning the previously mentioned heme-related pathological scenarios.

## Supporting information

**Supplementary Material:** The Molecular Basis of Transient Heme-Protein Interactions: Analysis, Concept and Implementation

## Abbreviations

ALAS: aminolevulinic acid synthase
Aβ: amyloid β
*cw*EPR: continuous-wave electron spin resonance spectroscopy
CP: cysteine-proline
HBM: heme-binding motif
HBP: heme-binding protein
HRM: heme-regulatory motif
MALDI-TOF: matrix-assisted laser desorption/ionization time of flight
PED: partial Edman degradation
rRaman spectroscopy: resonance Raman spectroscopy
SeqD-HBM: sequence-based detection of heme binding motifs
WESA: weighted ensemble solvent accessibility
