# Supplementary material for: The molecular basis of transient heme-protein interactions: analysis, concept and implementation
Source: Biosci Rep. 2019 Jan 30;39(1):BSR20181940. doi: 10.1042/BSR20181940 (PMC6356037; doi:10.1042/BSR20181940)
Supplement: Supplementary file 1 [file bsr20181940_Supp1.pdf]

Supplementary Material

The Molecular Basis of Transient Heme-Protein Interactions: Analysis, Concept and Implementation

Amelie Wißbrock[1], Ajay Abisheck Paul George[1],
Hans Henning Brewitz[1], Toni Kühl[1], and Diana Imhof[1]*

[1]*Pharmaceutical Biochemistry and Bioanalytics, Pharmaceutical Institute, University of Bonn, An der Immenburg 4, 53121 Bonn, Germany*

SeqD-HBM development

The current stand-alone version of *SeqD-HBM*, designed to be used both on Linux and Microsoft Windows based operating systems was written and tested in *Python 3.6.4* and *Python 2.7.15* on a Linux workstation running *Ubuntu 18.4* and on workstations running Windows 7 and Windows 10 operating systems. Parallel installations of *Python 2.X* and *3.X* versions are required on the same system to automatically post of the sequences to the WESA server for solvent accessibility predictions. This part of the logic was developed using hints from the WESA documentation for running batch jobs (http://pipe.sc.fsu.edu/PostHandler/WESA-PostHandler.htm). Windows users also need the *wget* program present as an executable in the working directory.

SeqD-HBM usage

With all of the programs and scripts correctly organized and the requirements fulfilled, *SeqD-HBM* can be run on the command line by issuing a command such as the following.

python SeqD-HBM_Linux_Win.py <input_fasta_file> default OR *python SeqD-HBM_Linux_Win.py <input_fasta_file> structure*

In the line above *<input_fasta_file>* is the name of the FASTA file with the sequence(s) and the *default* and *structure* arguments indicate the mode of operation. It must be noted that the input file must be placed in the same directory as the *SeqD-HBM* python script. The program can take multiple sequences given one below the other in a file as long as they are specified in the FASTA format. Even in the case of a single sequence, the program expects the sequence to be given in a input file with a header as per the FASTA format. In the *default* mode, the program automatically posts the sequence to the WESA server and attempts to fetch the solvent accessibility prediction once every two minutes until a successful output is obtained. The *default* mode will not process sequences more than 2000 amino acid residues long since this is a limit set by WESA.While using the program for a large number of sequences, it is recommended to redirect the output into a text file.

SeqD-HBM output

The current version of *SeqD-HBM* first checks all of the sequences in the file for junk or non-standard characters. The program only accepts the 20 standard amino acids as input which is the same case with WESA and informs the user if there are errors in the input file. The main output for each sequence is the table containing the predicted coordination site, the associated 9mer motif and the net charge on the motif. An additional column named “comment” provides useful hints to the user regarding each predicted motif. This includes special instances such as when the motif has a net charge of 0 or less but if the coordinating residue

is a cysteine or if the motif is a CP motif. Another message that can occur in the "comment" column of the output is hinting to the user the possible occurrence of disulfide bonds in the predicted motif when the sequence contains multiple cysteine residues.

SeqD-HBM testing

SeqD-HBM was tested for its accuracy and performance on different datasets. The performance of the program was tested on a set of ~600 protein sequences with an average sequence length of 135 amino acids. In the *structure* mode, where the WESA computation is skipped, the program took only 1.62 seconds to process the output for these proteins. Of course, since WESA uses five different machine learning algorithms, it does prove to be a performance bottleneck with respect to execution time especially while operating on a large set of sequences. However, this is an acceptable tradeoff considering the accuracy of the prediction when absolutely no structural information is available.

Test sequence

```
>sp|P06736|HLYC_ECOLX Hemolysin-activating lysine-acyltransferase HlyC
OS=Escherichia coli OX=562 GN=hlyC PE=1 SV=1
MNINKPLEILGHVSWLWASSPLHRNWPVSLFAINVLPAIQANQYVLLTRDDYPVAYCSWA
NLSLENEIKYLNDVTSLVAEDWTSGDRKWFIDWIAPFGDNGALYKYMRKKFPDELFRAIR
VDPKTHVGKVSEFHGGKIDKQLANKIFKQYHHELITEVKRKSDFNFSLTG
```

We use a 170 residue long sequence of the protein HlyC (Hemolysin-activating lysine-acyltransferase Uniprot ID P06736) from *Escherichia coli* as a test sequence to demonstrate the usage of *SeqD-HBM* in both its *default* and *structure* modes of operation. This protein has no experimentally determined structure available. *SeqD-HBM* used in with the *structure* mode took 0.05 seconds to execute and predict H23, C57, Y104, Y106, H126, H134, Y150, H151 and H152 as the potential heme binding sites. Running the same sequence in the default mode took 7.64 minutes (458.39 seconds) for the overall execution with the WESA computation but the prediction eliminated the residues C57, Y104 and Y150 as buried hence improving the accuracy by removing 3 false positives.
